# Role of Kv1 Potassium Channels in Regulating Dopamine Release and Presynaptic D2 Receptor Function

**DOI:** 10.1371/journal.pone.0020402

**Published:** 2011-05-27

**Authors:** Philippe Martel, Damiana Leo, Stephanie Fulton, Maxime Bérard, Louis-Eric Trudeau

**Affiliations:** Department of Pharmacology, Faculty of Medicine, Groupe de Recherche sur le Système Nerveux Central, Université de Montréal, Montréal, Québec, Canada; University of North Dakota, United States of America

## Abstract

Dopamine (DA) release in the CNS is critical for motor control and motivated behaviors. Dysfunction of its regulation is thought to be implicated in drug abuse and in diseases such as schizophrenia and Parkinson's. Although various potassium channels located in the somatodendritic compartment of DA neurons such as G-protein-gated inward rectifying potassium channels (GIRK) have been shown to regulate cell firing and DA release, little is presently known about the role of potassium channels localized in the axon terminals of these neurons. Here we used fast-scan cyclic voltammetry to study electrically-evoked DA release in rat dorsal striatal brain slices. We find that although G-protein-gated inward rectifying (GIRK) and ATP-gated (K_ATP_) potassium channels play only a minor role, voltage-gated potassium channels of the Kv1 family play a major role in regulating DA release. The use of Kv subtype-selective blockers confirmed a role for Kv1.2, 1.3 and 1.6, but not Kv1.1, 3.1, 3.2, 3.4 and 4.2. Interestingly, Kv1 blockers also reduced the ability of quinpirole, a D2 receptor agonist, to inhibit evoked DA overflow, thus suggesting that Kv1 channels also regulate presynaptic D2 receptor function. Our work identifies Kv1 potassium channels as key regulators of DA release in the striatum.

## Introduction

Dopamine (DA) release in the CNS is critical for motor control by basal ganglia circuits and a dysfunction of its regulation is thought to be implicated in adaptations of the brain in response to drugs of abuse as well as in diseases such as schizophrenia and Parkinson's. A number of control mechanisms regulating DA release have been identified. For example, DA has long been known to regulate its own release through the activation of autoreceptors [1]. The activation of D2 autoreceptors located on the soma and dendrites of DA neurons inhibits cell firing [2], [3], [4] and decreases somatodendritic DA release [5], [6], [7]. It can also activate DA reuptake [8], [9] and inhibit DA synthesis [10], [11], [12], [13]. The hyperpolarizing effect of somatodendritic D2 autoreceptors has been proposed to occur principally through activation of G-protein-gated inward rectifying K^+^ channels (GIRKs) [14], [15], [16]. Autoreceptors are also present on the axon terminals of DA neurons [17], [18]. Electrically-evoked DA release in the striatum can be inhibited by D2-type receptor agonists and enhanced by D2-type receptor antagonists [19], [20]. A specific role for the D2-short splice variant of the D2 receptor in this process was confirmed by the near absence of autoreceptor function in D2 knockout mice [21], [22], [23], [24], and the maintenance of D2-autoreceptor function in D2-long knockout mice [21] and in D3 knockout mice [25].

The critical role of somatodendritic GIRK channels in regulating DA release raises the question as to whether such channels or other types of potassium channels are also present on dopaminergic axon terminals in the striatum and are involved in regulating DA release. Although GIRK channels are usually not found on axon terminals [26], there is evidence for the presence of voltage-gated Kv-type K^+^ channels [27] and of K_ATP_ channels [28], [29]. For example, using a striatal slice preparation, Cass et al. showed that the wide-spectrum Kv channel blockers 4-aminopyridine (4-AP) and tetraethylammonium (TEA) enhance electrically-evoked [^3^H]DA release [27]. However, the Kv channel subtype that is targeted by 4-AP in the terminals of DA neurons is currently unknown. In the present work, we took advantage of selective Kv neurotoxins and fast-scan cyclic voltammetry in a rat striatal brain slice preparation to directly examine the role of Kv potassium channel subtypes in controlling electrically-evoked DA release. We find an important role of Kv1-type potassium channels and show in addition that these channels act as a gating mechanism to influence presynaptic D2 function.

## Methods

### Ethics Statement

All experiments were approved by the Université de Montréal's animal ethics committee (protocol #10-122). All efforts were made to minimize the number of animals used and their suffering.

### Brain slice preparation and solutions

Four to six weeks old male and female Sprague-Dawley rats were anesthetized with halothane and quickly decapitated. Coronal striatal brain slices of 300 µm (Bregma 1.70 to 0.48 mm) [30] were prepared with a VT1000S vibratome (Leica Microsystems Inc., Nussloch, Germany) in ice-cold (0 to 4°C) artificial CSF (ACSF) containing (in mM): 125 NaCl, 26 NaHCO_3_, 2.5 KCl, 2.4 CaCl_2_, 1.3 MgSO_4_, 0.3 KH_2_PO_4_ and 10 D-Glucose; adjusted to 300 mOsm/kg and saturated with 95% O_2_-5% CO_2_. Slices were then kept in ACSF at room temperature and allowed to recover for at least 1 hour. For recordings, slices were put in a custom-made recording chamber superfused with ACSF (1 ml/min) maintained at 32°C with a TC-324B single channel heater controller (Warner Instrument Inc., Hamden, CT, USA). All drugs and chemicals were obtained from Sigma-Aldrich Canada (Oakville, ON). Glibenclamide was purchased from Tocris (Ellisville, MO). All toxins were obtained from Alomone labs (Jerusalem, Israel). All drugs and toxins were kept frozen in individual aliquots and thawed just before use. The pH of solutions containing the K^+^ channel antagonists 4-AP and TEA was adjusted to 7.4 prior to use.

### Electrochemical recordings and electrical stimulation

Electrically-evoked, action potential-induced DA release was measured by fast-scan cyclic voltammetry (FSCV) using a 5 µm diameter carbon-fiber electrode placed into the dorsal striatum ∼100 µm below the surface. Carbon-fiber electrodes were constructed according to Kawagoe et al. [31]. Briefly, carbon fibers (Cytec Industries Inc., NJ, USA) approximately 5 µm in diameter were aspirated into ethanol-cleaned glass capillaries (1.2 mm ×0.68 mm, 4 inches long; A-M Systems, WA, USA). The glass capillaries were then pulled using a P-2000 micropipette puller (Sutter Instruments, Novato, USA), dipped into 90°C epoxy for 30 sec (Epo-Tek 301, Epoxy Technology, MASS, USA) and cleaned in hot acetone for 3 seconds. The electrodes were heated at 100°C for 12 hours and 150°C for 5 days. Prior to use, electrodes were cleaned with agitated isopropyl alcohol for 15 min to promote greater sensitivity [32]. Carbon fibers were cut using a n°11 scalpel blade under direct visualization to obtain maximal basal currents of 100 to 180 nA. Electrodes were finally selected for their DA sensitivity using *in vitro* calibration with 1 µM DA in ACSF.

**Figure 1:**
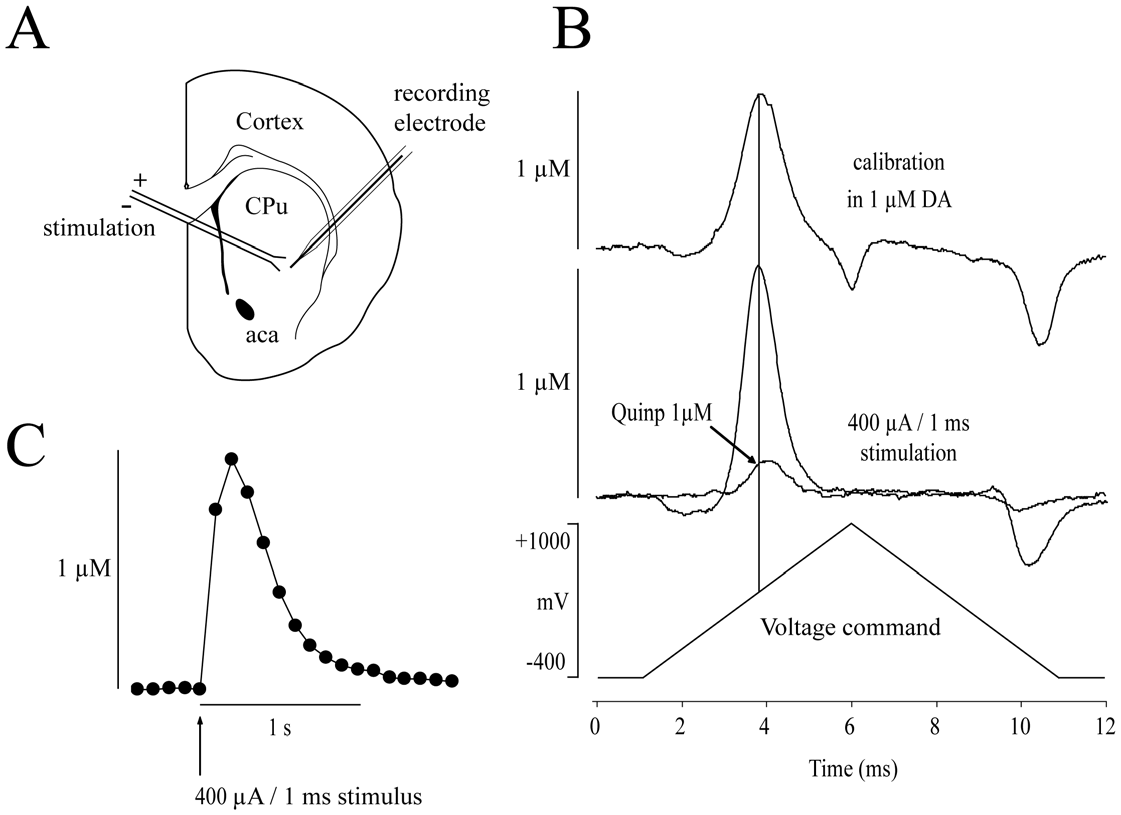
Fast-scan cyclic voltammetry in the rat dorsolateral striatum. (A) DA release was electrically-evoked with a bipolar stimulating electrode and recorded with FSCV using a 5 µm carbon-fiber recording electrode placed in the dorsolateral striatum. (B) Background-subtracted traces showing oxydo-reduction of DA recorded with a triangular voltage ramp (bottom trace). *In vitro* calibration with 1 µM DA (top recording) was used to convert the signal to DA concentrations. DA overflow was electrically-evoked with a 400 µA/1 ms single stimulation (middle trace). Quinpirole (1 µM) reduced DA overflow more than 75%. (C) Example of DA overflow kinetics. Each dot represents the peak amplitude of each oxidation traces obtained at intervals of 100 ms after a 400 µA/1 ms single stimulation. *Abbreviations*: aca, anterior commissure anterior part; CPu, Caudate putamen; Quinp, Quinpirole.

The potential of the carbon fiber electrode was scanned at a rate of 300 V/s according to a 10 ms triangular voltage wave (−400 to 1000 mV vs Ag/AgCl) with a 100 ms sampling interval using an Axopatch 200B amplifier (Axon Instruments, Union City, CA) or a Warner PC-501A (Warner Instrument Inc., Hamden, CT, USA) (Fig. 1B). Data were acquired using a DigiData 1200B analog to digital board converter (Axon Instruments) connected to a Compaq Pentium III personal computer using Clampex 9.2 (Axon Instruments). Single pulse stimulations (400 µA; 1 ms) were generated by a S-900 stimulator (Dagan Corporation, Minneapolis, MN) every 2 min to electrically evoke DA release in the dorsal striatum. The tips of the bipolar electrode (Plastics One, Roanoke, VA) were separated by approximately 100 to 150 µm and were gently placed on the surface of the slice using a micromanipulator. Under these conditions, DA release was tetrodotoxin-sensitive and Ca^2+^ dependent (data not shown).

### Data analysis

Channel blockers were superfused for 30 min, except where otherwise stated. Quinpirole was applied by superfusion for 4 min and the effect of this D2 agonist was evaluated at its peak. This short quinpirole application time may lead to underestimation of maximal effects and apparent IC50, but provides a reproducible effect. Control experiments with quinpirole alone were performed in parallel to experiments evaluating the effect of blockers. The effects of quinpirole in the presence of the various blockers were always compared to control experiments performed during the same series of experiments. In experiments in which K^+^ channel blockers were applied, a stable 10 min control period was first obtained followed by 30 min bath application of the blockers. The effect of drugs and toxins on DA release was determined by normalizing values against their own control period which represented the first 10 min of stable recording before drug application. Statistical analyses were conducted by comparing the means of the last 10 min of blocker application to the mean of the last 10 min of control recordings. Results are shown as mean ± SEM. The sample size is shown as n = (*x*;*y*), where *x* refers to the number of slices and *y* the number of animals. Only one experiment was performed on each slice. Unpaired t-tests were used when comparing two groups while one-way ANOVAs with *post hoc* comparisons (Tukey test) were used when comparing multiple groups of data. Significance levels used in figures are shown as: *single asterisk* (*)  =  p<0.05, *double asterisks* (**)  =  p<0.01 and *triple asterisks* (***)  =  p<0.001.

## Results

### Broad-spectrum potassium channel blockers enhance DA release

**Figure 2:**
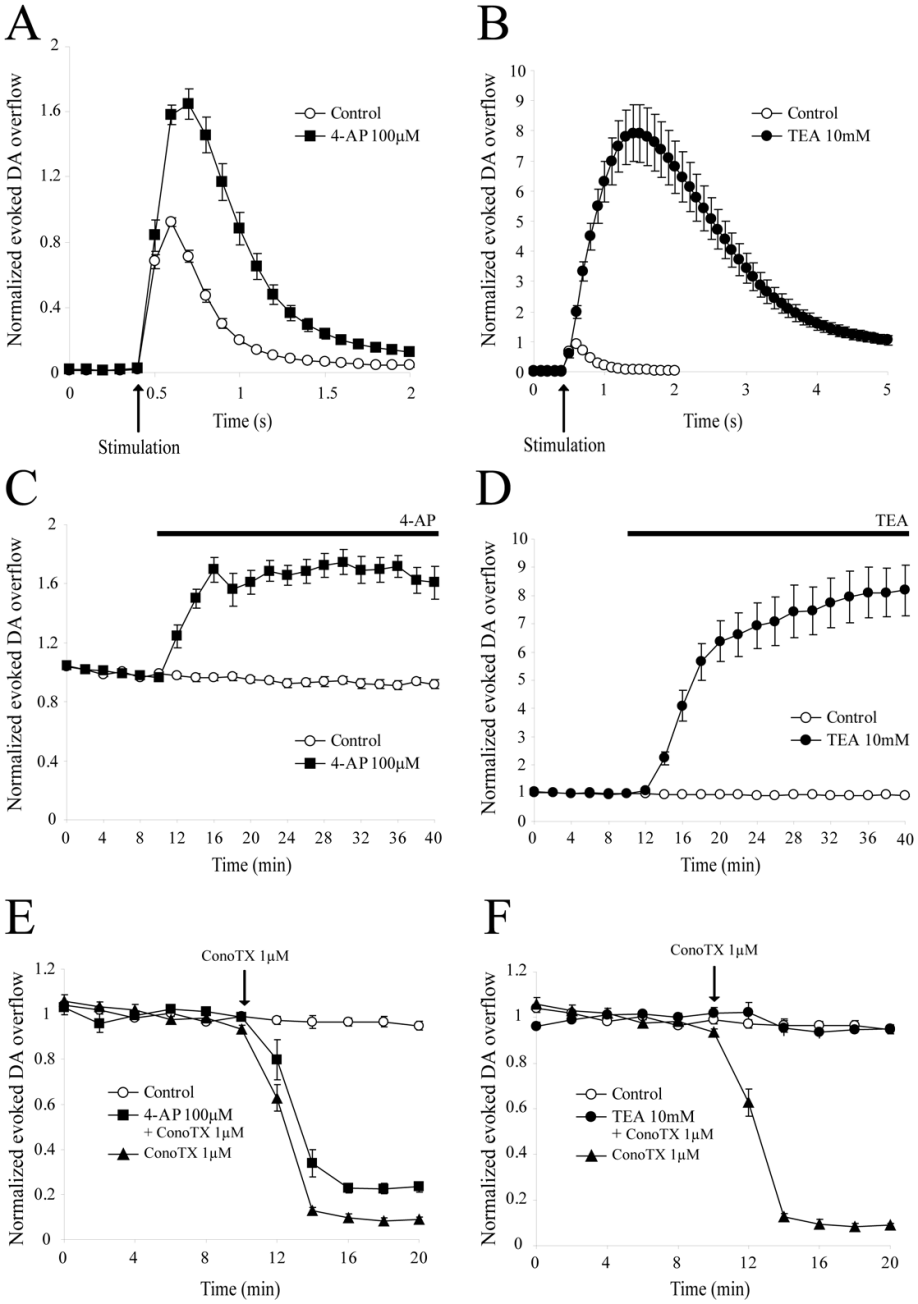
Effect of broad-spectrum potassium channel blockers on evoked DA overflow. (A) A 30 min perfusion of the broad-spectrum potassium channels blocker 4-AP (100 µM) (n = 10;4) increased DA overflow kinetics compared to control (n = 8;6). Data were normalized to the first 6 recordings (10 min) of their respective control period (means ± SEM). (B) A 30 min perfusion of the broad-spectrum potassium channels blocker TEA (10 mM) (n = 10;3) drastically increased DA overflow kinetics compared to control (n = 8;6). Data were normalized to the first 6 recordings (10 min) of their respective control period (means ± SEM). (C) Time-course of the effect of a 30 min application of 100 µM 4-AP (n = 10;4) in comparison to control (n = 8;6). DA was electrically evoked at intervals of 2 min. Each group is normalized to the first 6 recordings (10 min) of its respective control period and graphically plotted against time (means ± SEM). After 10 min of stable recordings, 4-AP was superfused for 30 min. (D) Time-course of the effect of a 30 min application of 10 mM TEA (n = 10;3) in comparison to control (n = 8;6). (E) After a 30 min application of 100 µM 4-AP, 1 µM ConoTX was applied and caused a ∼ 90% decrease in evoked DA release. (F) After a 30 min application of 10 mM TEA, 1 µM ConoTX was applied and failed to inhibit DA overflow. Abbreviations: 4-AP, 4-aminopyridine; TEA, tetraethylammonium; ConoTX, ω-conotoxin GVIA.

DA release was electrically-evoked with single pulses every 2 min in striatal brain slices from 4 to 6 weeks old Sprague-Dawley rats and measured using FSCV at 300 V/S (Fig. 1A and 1B). Evoked DA overflow in control conditions reached ∼1 µM (Fig. 1C). The detection limit due to experimental noise was approximately 0.06 µM (n = 19;7). While differences in the nigrostriatal pathway of female and male rats have been previously reported [33], [34], we didn't find any statistical difference in the maximal amount of DA release evoked by single pulse in males (1.04±0.09 µM; n = 23) and females (0.94±0.08 µM; n = 28) (data not shown; p>0.05). Recovery of extracellular DA levels, reflecting mostly reuptake, was rapid, requiring no more than 1 sec (Fig. 1C). Evoked DA overflow was stable over time with a mean rundown of 6.9±2.4% (n = 8;6) over a period of 40 min of control recording (see control trace in Fig. 2C).

Following this validation of our experimental conditions, we next evaluated the role of potassium channels using 4-AP and TEA, two broad-spectrum potassium channel blockers (Fig. 2). After a 30 min application of 100 µM 4-AP, DA overflow increased by 68.0±8.3% (n = 10;4) (Fig. 2A, 2C). At 1 mM, 4-AP rapidly increased DA overflow by ∼80%, but a significant rundown appeared, and after 30 min, DA overflow was maximally increased by 43.4±5.6% (data not shown, n = 7;3). At these two doses, the effect of 4-AP was statistically different from the basal rundown (p<0.001 for both) and the difference between the doses was statistically significant (p<0.05). At a dose of 10 mM, 4-AP was detrimental to slice survival, as revealed by the rapid rundown of electrically-evoked DA overflow (data not shown, n = 2;1). Considering toxicity at 10 mM and a rundown effect at 1 mM, all further experiments were thus performed with 100 µM 4-AP.

At 1 mM, TEA increased DA overflow by 12.9±6.0%, which was not different from basal rundown (data not shown, n = 6;3, p>0.05). Surprisingly, at 10 mM, TEA caused a very large, yet stable increase of 792.9±88.0% (n = 10;3, p<0.001) (Fig. 2B, 2D). This effect is not likely due to a change in osmolarity, since addition of 20 mOsm of sucrose in ACSF had no effect on electrically-evoked DA overflow (data not shown). Moreover, this effect was not due to an increase in electrode sensitivity for DA, because *in-vitro* calibration using 1 µM DA in the presence of 10 mM TEA did not reveal any change in comparison to control conditions without TEA (data not shown).

A potential explanation of the extensive increase in evoked DA release induced by 10 mM TEA is that enhanced depolarization of axon terminals may occur under such conditions, perhaps leading to the recruitment of additional synaptic and extra-synaptic voltage-gated Ca^2+^ channels. To address this issue, we first determined the subtype of voltage-gated Ca^2+^ channels required for electrically-evoked DA release under our experimental conditions. Although 20 µM nifedipine, a L-type Ca^2+^ channel blocker, and 200 nM ω-agatoxin IVA, a blocker which is specific for P/Q-type Ca^2+^ channels at this concentration, did not significantly reduce DA release (12.3±1.3% decrease; n = 6;3; p>0.05 and 14.3±3.0% decrease; n = 6;2; p>0.05, respectively) (results not shown), the N-type Ca^2+^ channel blocker ω-conotoxin GVIA (ConoTX) almost totally blocked DA release at 1 µM (91.7±1.2% decrease, ; n = 5;2 ; p<0.001) (Fig. 2E). We next evaluated the effect of ConoTX after pre-application of 100 µM 4-AP or 10 mM TEA. We reasoned that if non-N-type extrasynaptic Ca^2+^ channels were recruited, this should result in an increase in the fraction of the release that is resistant to ConoTX block. After a 30 min pre-application of 100 µM 4-AP, ConoTX (1 µM) produced a slightly reduced but still strong decrease of DA release by 74.6±2.2% (n = 6;3), which was statistically different from the effect of ConoTX alone (p<0.001) (Fig. 2E). However, in the presence of 10 mM TEA, ConoTX (1 µM) failed to significantly inhibit evoked DA overflow as 91.4±2.9% of the signal still remained (n = 6;2) (Fig. 2F). Overall, these data show that although 4-AP increases DA release by about 70%, this extra release remains largely dependent on N-type calcium channels. On the contrary, the large increase produced by TEA appears to be dependent on additional, perhaps extra-synaptic, Ca^2+^ channels that are insensitive to the N-type Ca^2+^ channel antagonist and that might be recruited by non-physiological depolarization of axon terminals.

### Kv subunit blockers increase electrically-evoked DA overflow

**Figure 3:**
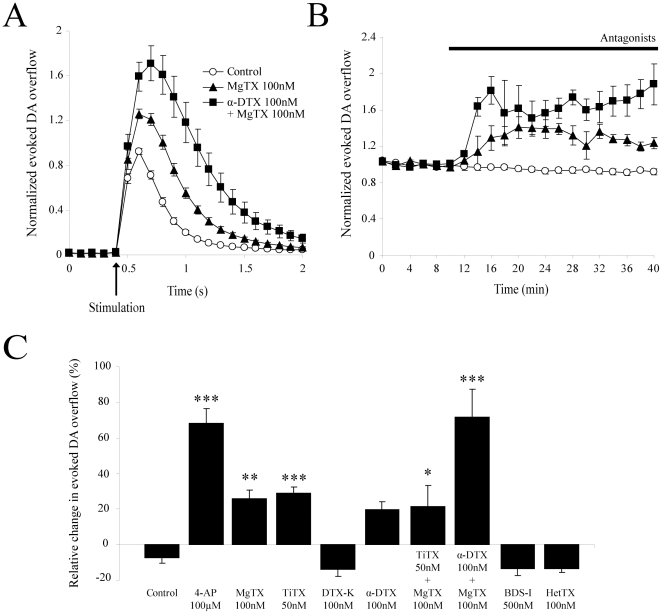
Effect of selective Kv potassium channel subunit blockers on evoked DA overflow. (**A**) Summary traces showing the average electrically-evoked DA overflow in response to single electrical pulses after 30 min perfusion with 100 nM MgTX (n = 5;3) or a combination of both 100 nM MgTX and 100 nM α-DTX (n = 4;2), in comparison to control (n = 8;6). Data were normalized to the first 6 recordings (10 min) of their respective control period (means ± SEM). The effect of other Kv subunits blockers is omitted for clarity. (**B**) Time-course of the experiment shown in *A*. DA was electrically evoked at intervals of 2 min. Each group is normalized to the first 6 recordings (10 min) of its respective control period and graphically plotted against time (means ± SEM). After 10 min of stable recordings, drugs were superfused for 30 min. Apparent equilibrium was reached for all Kv channel blockers after 20 min of application. The effect of others Kv channel blockers is omitted for clarity. (**C**) Histogram showing the mean increase in electrically-evoked DA overflow during the last 10 min of application of several Kv potassium channel blockers: control (n = 8;6), 100 µM 4-AP (n = 10;4), 100 nM MgTX (n = 5;3), 50 nM TiTX (n = 6;2), 100 nM DTX-K (n = 6;3), 100 nM α-DTX (n = 6;4), 50 nM TiTX + 100 nM MgTX (n = 4;3), 100 nM α-DTX + 100 nM MgTX (n = 4;2), 500 nM BDS-I (n = 6;2) and 100 nM HetTX (n = 6;2). * p<0.05, ** p<0.01, *** p<0.001. *Abbreviations*: 4-AP, 4-aminopyridine; α-DTX, α-dendrotoxin; MgTX, rMargatoxin; TiTX, rTityustoxin Kα; DTX-K, dendrotoxin-K; BDS-I, blood depressing substance I; HetTX, rHeteropodatoxin-2.

At the concentration we used, 4-AP blocks several voltage-dependent potassium channels containing Kv1.x and Kv3.x subunits [35]. However, considering previous reports suggesting the presence of Kv1-family potassium channels in various axon terminals in the CNS [36], we hypothesized that Kv1 channels could play a particularly important role. To discriminate between the various Kv subtypes, we used several toxins shown to specifically inhibit different Kv channels (Fig. 3). At 100 nM, r-Margatoxin (MgTX), a blocker of Kv1.3 channels [37], increased DA levels by 25.8±4.9% (n = 5;3, p<0.01) (Fig. 3A, 3B, 3C). At 50 nM, r-Tityustoxin Kα (TiTX), a specific Kv1.2 subtype blocker [38], increased DA levels by 29.1±3.2% (n = 6;2, p<0.001) (Fig. 3C). At 100 nM, dendrotoxin-K (DTX-K), a specific Kv1.1 subtype blocker [39], caused no significant change in DA levels compared to rundown (14.0±3.9% decrease, n = 6;3, p>0.05). Finally, at 100 nM, α-Dendrotoxin (α-DTX), a specific Kv1.1, 1.2 and 1.6 subtype blocker [39], produced a variable increase in DA levels which did not reach statistical significance (15.5±5.6% increase, n = 6;4, p = 0.08).

To narrow down on which Kv1 subtypes are important for regulating axonal DA release, we also tested some toxin combinations. A 30 min application of both 50 nM TiTX and 100 nM MgTX increased DA levels by 21.6±11.5% (n = 4;3, p<0.05) (Fig. 3C), while a 30 min application of both α-DTX (100 nM) and MgTX (100 nM) increased DA levels by 71.4±15.7% (n = 4;2, p<0.001) (Fig. 3A, 3B, 3C). Together, these data suggest a role for Kv1.2, Kv1.3 and Kv1.6 in regulating DA release.

Because 4-AP can also block some Kv3.x-containing potassium channels, we also evaluated the effect of blood depressing substance I (BDS-I), a blocker of Kv3.4 in the low nanomolar range and in addition of Kv3.1 and Kv3.2 subunits at higher doses [40]. At 500 nM, BDS-I caused no significant change in DA levels compared to rundown (13.3±4.0% decrease) (n = 6;2, p>0.05) (Fig. 3C). As a negative control, we also evaluated the effect of 100 nM, r-Heteropodatoxin-2 (HetTX), a specific blocker of Kv4.2 channels, a K^+^ channel subunit known to be usually restricted to the somatodendritic compartment of neurons and that is not expressed by DA neurons [41]. HetTX caused no significant change in DA levels compared to rundown (13.4±2.2% decrease) (n = 6;2, p>0.05) (Fig. 3C).

### Kv1 channels regulate D2-mediated inhibition of DA release

Considering the important role of Kv1 channels in regulating DA release, identified in the present report, and the existence of previous reports suggesting reduced autoreceptor function in the presence of 4-AP [18], [27], we next hypothesized that Kv1 channels act as mediators of presynaptic D2 function or as a gating mechanism controlling the effectiveness of this receptor, perhaps through regulation of terminal polarity.

**Figure 4:**
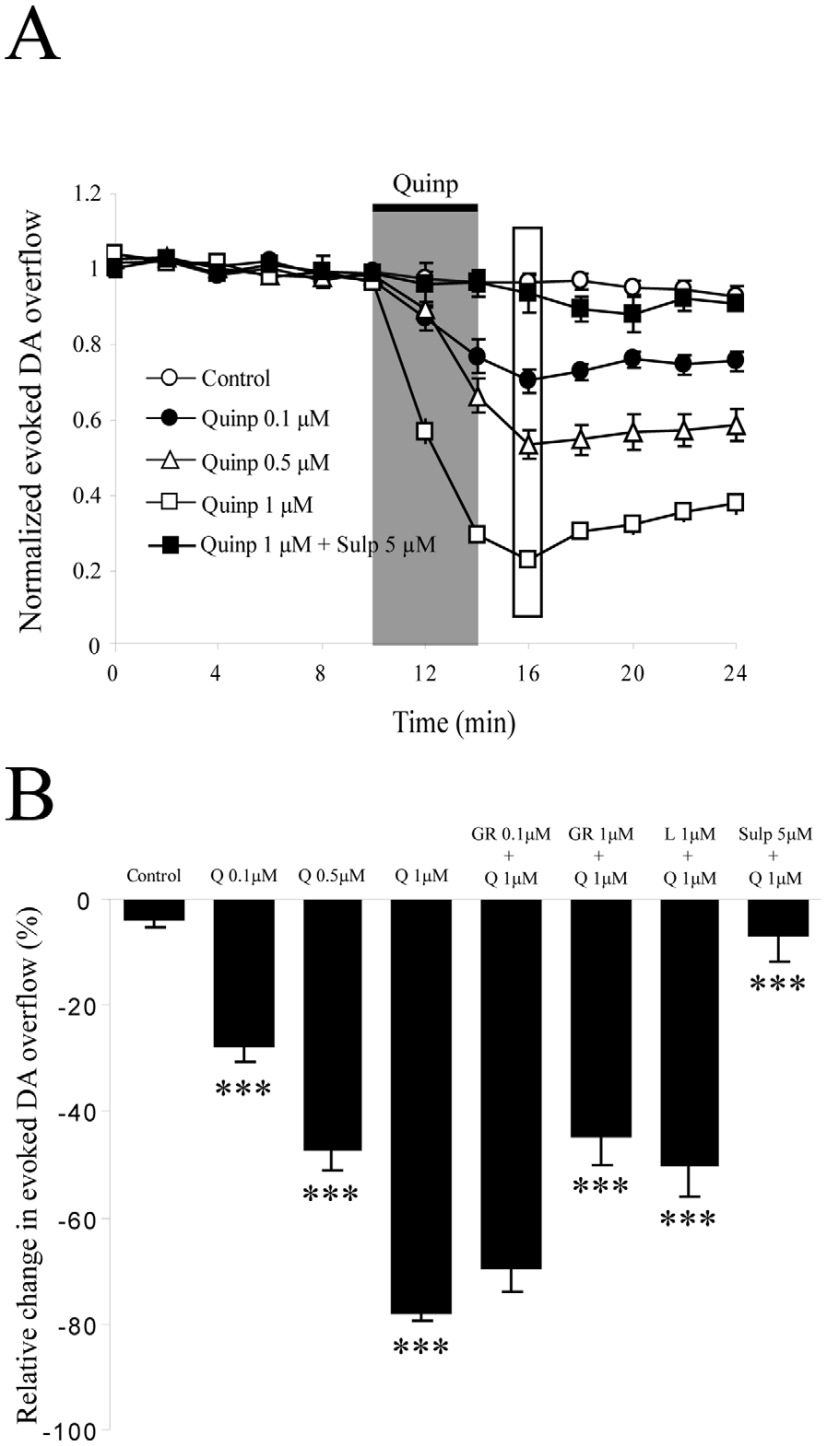
D2 receptor activation dose-dependently inhibits dopamine overflow. A) DA was electrically-evoked at intervals of 2 min. The peak amplitude of DA overflow is normalized to the average amplitude of the first 6 recordings (10 min) of the control period and graphically plotted against time (means ± SEM). The control trace (n = 8;6) shows that evoked DA overflow was stable over time. Quinpirole was applied for 4 min (represented by the gray bar) at 3 different doses. Drug effects were measured at their peak (rectangle at 16 min). Sulpiride (5 µM) blocked entirely the effect of quinpirole. (B) Histogram showing the peak effect of quinpirole on DA overflow. A one-way ANOVA was used to compare groups. The effect of quinpirole at 0.1 µM (n = 5;4), 0.5 µM (n = 7;4) and 1 µM (n = 27;18) was significant. The D3 receptor subtype blocker, GR103,691, only reduced quinpirole effect at the non-selective dose of 1 µM (n = 4;2), while its effect was non-significant at 100 nM (n = 6;2). Used at 1 µM, L741,626, a D2 subtype selective blocker, significantly reduced the effect of quinpirole (n = 6;2). Sulpiride (5 µM), a broad-spectrum D2-family receptor antagonist, complety blocked the effect of quinpirole (n = 3;2). *** p<0.001 *Abbreviations*: Q, Quinpirole; Quinp, Quinpirole; GR, GR103,691; L, L741,626, Sulp, Sulpiride.

We first confirmed that, as expected, the D2 agonist quinpirole decreased electrically-evoked DA release in a dose-dependent manner (Fig. 4A and 4B). A 4 min application of 0.1 µM, 0.5 µM or 1 µM quinpirole decreased DA levels by 27.5±2.7% (n = 7;5, p<0.001), 46.7±3.8% (n = 7;4, p<0.001) and 77.5±1.7% (n = 27;18, p<0.001), respectively. Again, we found no sex difference in the effect of 1 µM quinpirole in males (75.4±1.5% decrease; n = 11;7) and females (73.7±3.4% decrease; n = 6;3) (data not shown; p>0.05). Because quinpirole can bind to the D3 receptor subtype in addition to the D2 subtype, we evaluated whether the effect of quinpirole was reduced by GR103,691, a D3-subtype preferring antagonist and L741,626, a D2-subtype preferring antagonist. We found that at a concentration of 100 nM, known to occupy a majority of D3 receptors (Murray et al., 1995; Audinot et al., 1998), a 20 min pre-application of GR103,691 failed to significantly reduce the effect of 1 µM quinpirole on DA overflow evoked by single pulses (69.2±4.7% decrease; n = 6;2; p>0.05) (Fig. 4B). At a concentration of 1 µM, which would be predicted to block a subset of D2 receptors in addition to D3 receptors, GR103,691 caused a significant reduction in the effect of quinpirole (44.3±5.5% decrease; n = 4;2; p<0,001) (Fig. 4B). In comparison, at a concentration of 100 nM, known to occupy a significant proportion of D2 but not D3 receptors (Bowery et al., 1996; Pillai et al., 1998), L741,626 significantly reduced the effect of quinpirole (49.8±6.0% decrease; n = 6;2), which was different from the effect of quinpirole alone; p<0.001) (Fig. 4B). Complete block of the effect of quinpirole could be achieved using sulpiride (5 µM), a broad-spectrum D2-family receptor antagonist (6.6±5.1% decrease; n = 3;2, p<0.001) (Fig. 4A). However, none of the three receptor antagonists by themselves had a significant effect on DA overflow: they caused a decrease of 6.2±2.3% (sulpiride 5 µM), 11.3±1.5% (GR103,691 1 µM) or 16.4±3.4% (L741,626 100 nM) after 10 min, which was not statistically different from the basal rundown (data not shown; p>0.05). These results indicate that electrically-evoked DA overflow under our conditions can be inhibited by a D2 receptor-mediated mechanism in a dose-dependent manner under conditions where there is no detectable basal activation of these receptors following single pulse stimulation. These observations confirm previous findings [5], [25], [42].

**Figure 5:**
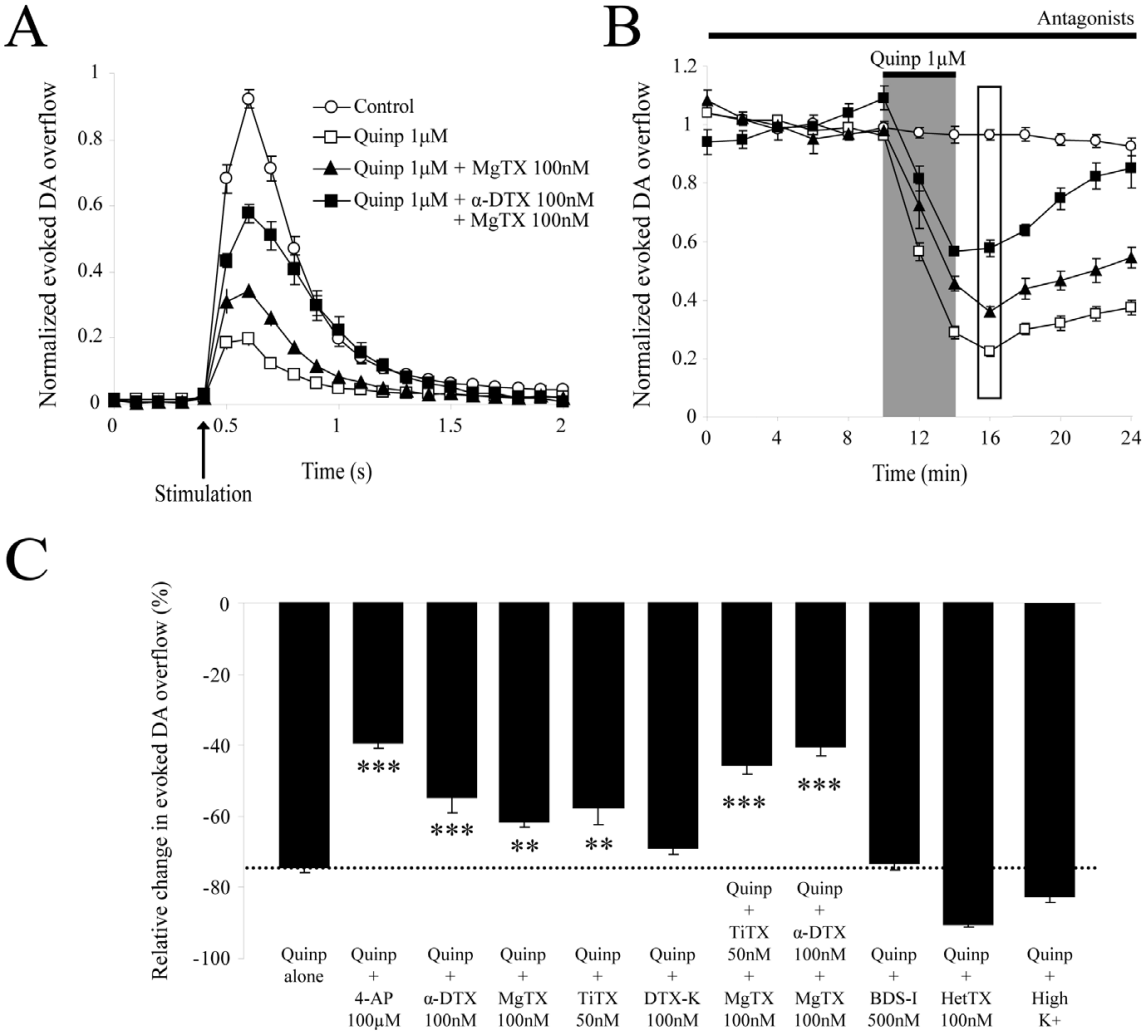
Regulation of D2-mediated inhibition of DA release by Kv1 potassium channels. (A) Applied alone for 4 min, 1 µM quinpirole (n = 27;18) induced a large inhibition of evoked DA overflow compared to control (n = 8;6). Pre-application (30 min) of 100 nM MgTX (n = 5;3) or a combination of both 100 nM MgTX and 100 nM α-DTX (n = 4;2) reduced the effect of quinpirole. Data were normalized to the first 6 recordings (10 min) of their respective control period (means ± SEM). The effect of others Kv subunits blockers is omitted for clarity. (B) Time-course of the experiment shown in *A*. DA was electrically-evoked at intervals of 2 min. Each group was normalized to the first 6 recordings (10 min) of its respective control period (means ± SEM). The maximal effect of quinpirole was detected after 6 min (represented by the rectangle). (**C**) Histogram showing the ability of potassium channel blockers selective for various Kv subtypes to reduce the effect of quinpirole (1 µM) on evoked DA overflow: quinpirole alone (n = 27;18, represented by the black doted bar), 4-AP (n = 6;2), 100 nM α-DTX (n = 6;4), 100 nM MgTX (n = 5;3), 50 nM TiTX (n = 6;2), 100 nM DTX-K (n = 6;3), 50 nM TiTX + 100 nM MgTX (n = 6;4), 100 nM α-DTX + 100 nM MgTX (n = 6;3), 500 nM BDS-I (n = 6;2), 100 nM HetTX (n = 6;2) and high concentration of potassium (n = 4;2). * p<0.05, ** p<0.01, *** p<0.001. *Abbreviations*: K^+^, potassium; 4-AP, 4-aminopyridine; α-DTX, α-dendrotoxin; MgTX, rMargatoxin; TiTX, rTityustoxin Kα; DTX-K, dendrotoxin-K; BDS-I, blood depressing substance I; HetTX, rHeteropodatoxin-2; quinp, quinpirole.

We next evaluated the role of K^+^ channels in D2-mediated inhibition of DA overflow. After a 30 min pre-application of 100 µM 4-AP, quinpirole (1 µM) decreased DA release by only 41.1±1.5% (n = 6;2) (Fig. 5C). This effect was statistically different from that induced by quinpirole alone (p<0.001). After a 30 min pre-application of 1 mM or 10 mM TEA, quinpirole (1 µM) decreased DA release by 62.9±3.4% (n = 6;3) and 12.1±3.9% (n = 6;2) respectively (data not shown), a significant reduction in comparison to the effect of quinpirole alone (p<0.01 and p<0.001 respectively).

To narrow down the role of different Kv subunits in regulating D2-mediated inhibition of DA release, we measured the effect of quinpirole (1 µM) after a 30 min pre-application of subtype-specific potassium channel toxins (Fig. 5). After pre-treatment with 100 nM α-DTX, a Kv1.1, 1.2 and 1.6 blocker, quinpirole decreased DA release by 53.2±3.9%, an effect that was statistically different from the effect of quinpirole alone (n = 6;4, p<0.001). In the presence of 100 nM MgTX, a Kv1.3 blocker, quinpirole decreased DA release by 64.1±1.8%, which was again statistically different from the effect of quinpirole alone (n = 5;3, p<0.01). In the presence of 50 nM TiTX, a kv1.2 blocker, quinpirole decreased DA release by 60.1±5.1%, which was statistically different from the effect of quinpirole alone (n = 6;2, p<0.01). In the presence of 100 nM DTX-K, a Kv1.1 blocker, quinpirole decreased DA release by 71.7±2.1%, which was not statistically different from the effect of quinpirole alone (n = 6;3, p>0.05).

In the presence of both 50 nM TiTX and 100 nM MgTX, quinpirole decreased DA release by 46.1±2.7%, which was statistically different from the effect of quinpirole alone (n = 6;4, p<0.001). Finally, with a 30 min pre-application of a combination of 100 nM MgTX and 100 nM α-DTX, quinpirole decreased DA release by 43.9±2.8%, an effect that was statistically different from the effect of quinpirole alone (n = 6;3; p<0.001). As expected, pre-application of 500 nM BDS-I or 100 nM HetTX did not cause any significant change in quinpirole-induced inhibition of DA release (76.3±2.1% decrease, n = 6;2 and 94.2±0.8% decrease, n = 6;2; p>0.05 for both). Overall, these results suggest that Kv1.2, Kv1.3 and Kv1.6 potassium channel subtypes can regulate D2-mediated inhibition of DA release while Kv1.1, Kv3.1, Kv3.2, Kv3.4 and Kv4.2 are unlikely to be involved.

Although unlikely in the case of voltage-gated channels such as those of the Kv1 family, potassium channel blockers could theoretically depolarize axon terminals, which could perhaps lead to partial inactivation of sodium or calcium channels. This could represent an alternative explanation of our finding that potassium channel blockers reduce the effect of the D2 agonist quinpirole. To evaluate this possibility, we exposed slices to ACSF containing 8.5 mM extracellular potassium (instead of 2.8 mM), thus directly causing a modest depolarization of dopaminergic terminals (estimated by patch-clamping to be of a magnitude of less than 5 mV; results not shown). Validating the effectiveness of the depolarization, we noted that after 20 min of elevated potassium, single-pulse evoked DA overflow was reduced by 37.1±10.4%, which was statistically more than the basal rundown (n = 4;2; p<0.001). However, under these conditions, the ability of quinpirole to reduce DA overflow was not significantly reduced (85.0±3.2% decrease; n = 4;2; p>0.05). These results argue that potassium channel blockers do not simply reduce the effect of quinpirole through a non-specific depolarization of terminals.

### Evaluation of the role of GIRK and K_ATP_ channels in regulating DA release

**Figure 6:**
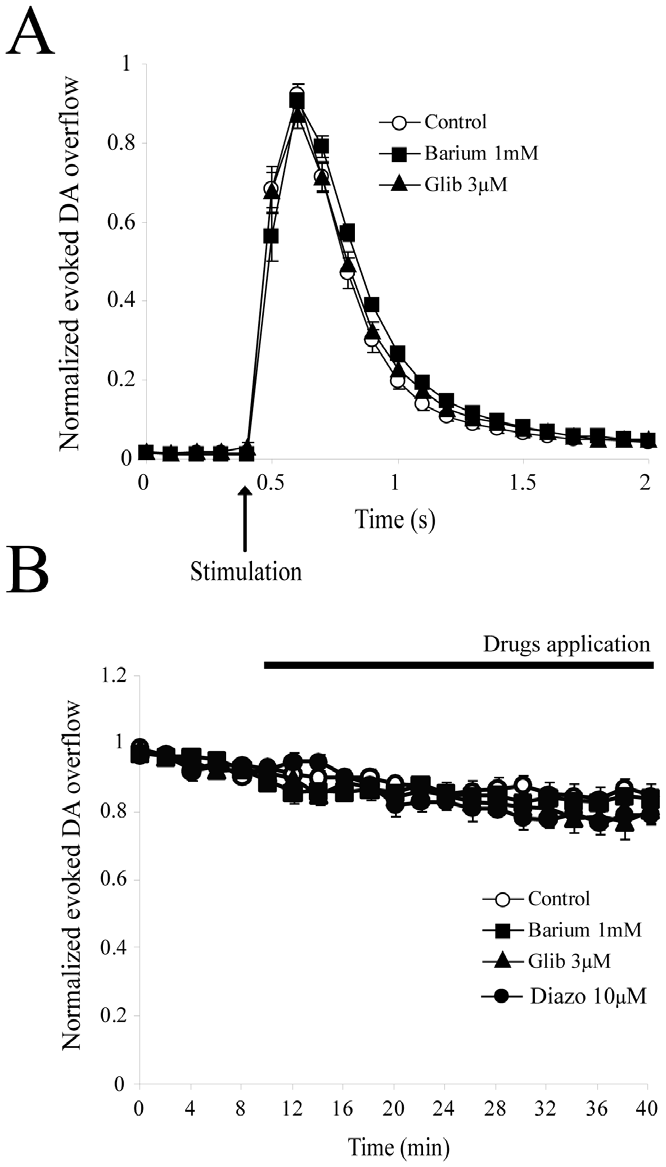
Evaluation of the role of GIRK and KATP channels in regulating DA release. (A) Effect of 30 min perfusion of the GIRK blocker barium (1 mM) (n = 7;3) or the KATP blocker glibenclamide (3 µM) (n = 7;4) on DA overflow kinetics compared to control (n = 8;6). Data were normalized to the first 6 recordings (10 min) of their respective control period (means ± SEM). No significant effect was noted. (B) Time-course of the experiment shown in *A*. DA was electrically evoked at intervals of 2 min. Each group was normalized to the first 6 recordings (10 min) of its respective control period (means ± SEM). After a 10 min stabilization period, blockers were applied for 30 min. No significant effect of these two antagonists was noted. *Abbreviations*: Glib, glibenclamide; Diazo, diazoxide.

Considering that even a broad-spectrum Kv potassium channel blocker such as 4-AP only blocked approximately 50% of the effect of quinpirole on evoked DA overflow, it is possible that other classes of potassium channels are also involved. Considering the presence of GIRK-like and K_ATP_ potassium channels in the somatodendritic compartment of DA neurons and their regulation through D2 receptors [43], [44], we next evaluated the effect of barium, a blocker of GIRK channels, and of glibenclamide, a blocker of K_ATP_ channels. Applied alone, 1 mM barium decreased evoked DA overflow by 7.1±2.2% (Fig. 6), which was not statistically different from spontaneous rundown (n = 7;3; p>0.05). Glibenclamide (3 µM) gave similar results (Fig. 6); a rundown of 11.6±3.4%, which was not statistically different from spontaneous rundown (n = 7;4; p>0.05). Moreover, a K_ATP_ channels opener, diazoxide (30 µM), had no significant effect on evoked DA overflow (decrease of 13.6±1.6%, which was not statistically different from spontaneous rundown) (Fig. 6B; n = 6;2; p>0.05).

We also evaluated the effect of the D2 agonist quinpirole in the presence of these two antagonists. After a 30 min pre-application of 1 mM barium, quinpirole (1 µM) decreased DA release by 78.2±4.9%, which was not statistically different from the effect of quinpirole alone (data not shown, n = 7;3; p>0.05). In the presence of 3 µM glibenclamide, quinpirole (1 µM) decreased DA release by 64.5±7.6%, which represented a small but statistically significant decrease compared to the effect of quinpirole alone (data not shown, n = 6;3; p<0.05). We conclude that while GIRK channels do not play any apparent role in regulating basal, electrically-evoked DA release under our experimental conditions, K_ATP_ channels may contribute to a minor proportion of the D2-mediated inhibition of DA release.

## Discussion

A better understanding of the regulatory mechanisms and ionic channels that control DA release is of major interest. Little is presently known concerning the potassium channels that are present in the axon terminals of DA neurons in the striatum. Here we provide an evaluation of the role of Kv1 potassium channels in the regulation of DA release. We show that Kv1.2, Kv1.3 and Kv1.6 are likely to be key players in regulating axonal DA release. We also show that these channels act as mediators or regulators of presynaptic D2 receptor function.

### Potassium channel regulation of DA release

A number of potassium channels have been demonstrated to be present in the somatodendritic compartment of DA neurons. This includes GIRKs [43], [45], [46], [47], [48], Ca^2+^-activated potassium channels [49], [50], K_ATP_ channels [28], [29] and various voltage-dependent potassium channels mediating rapidly-inactivating and delayed potassium current [51]. For example, D2 receptors can activate potassium conductances in the somatodendritic compartment of DA neurons that are compatible with the properties of GIRK channels (Lacey et al. 1987, Uchida et al. 2000). In cell lines and in brain slices, D2 receptors have been confirmed to couple to the GIRK2 potassium channels, the only GIRK subtype expressed by DA neurons [43], [45], [46], [47], [48]. It has been generally assumed that GIRK channels are also present in the axon terminals of DA neurons and that they are required for D2 autoreceptor function. Despite the fact that this possibility has not been directly examined, it is unlikely that this is the case, because in the mesencephalon as well as in many brain regions, GIRKs are mainly expressed somatodendritically and are not involved in terminal regulation [26], [52], [53], [54]. Compatible with this hypothesis, we found that blocking GIRK channels with barium failed to enhance electrically-evoked DA overflow. In addition, barium failed to reduce the ability of the D2 agonist quinpirole to inhibit DA overflow in the striatum. Within this context however, it is puzzling that tertiapin-Q, a selective antagonist of GIRK1/3 channels enhances electrically-evoked DA release in the guinea pig striatal slice preparation [28]. This could perhaps reflect an age- or species-difference in GIRK channel subtype expression or be due to the ability of tertiapin-Q to also block other potassium channels such as Ca^2+^-activated potassium channels [55]. Considering the questionable selectivity of this peptide, we have not included this antagonist in the present study. However, we have previously determined that tertiapin-Q fails to reduce the effect of quinpirole on electrically-evoked DA overflow in rat striatal slices (single-pulse evoked DA overflow was reduced by 68.7±7.4%, n = 8, p>0.05; Martel & Trudeau, unpublished observations).

Our results obtained with Kv blockers provide strong data in favor of the presence of Kv1 channels and in particular Kv1.2, Kv1.3 and perhaps Kv1.6 in the axonal terminals of DA neurons. Our findings are compatible with those of a recent study performed in mice and also suggesting the implication of Kv1.2 and Kv1.6 in the regulation of DA release [56]. The direct enhancement of evoked DA release by MgTX in the present study suggests the involvement of Kv1.3 channels [37], [57], [58]. Together with the lack of effect of the Kv1.1 specific toxin DTX-K and the effectiveness of TiTX, a blocker of Kv1.2, these results suggest that Kv1.2 and Kv1.3 are particularly important. The reason for the lack of additive effect of MgTX and TiTX on evoked DA overflow and the variable effect of the mixed Kv1.1, Kv1.2 and Kv1.6 blocker α-DTX are unclear. However, the additive effects of α-DTX and MgTX provides additional support for the involvement of Kv1.2, Kv1.3 and perhaps Kv1.6 in regulating DA release [36], [59], [60], [61], [62]. Considering the previous demonstration that Kv1 channels can form heteromers in the brain [63] and in peripheral tissues [64], one possibility is that Kv1 channels in DA neuron terminals form heteromers. For example, if Kv1.2/Kv1.3 heteromers are formed, these could in principle be blocked by either TiTX or MxTX, explaining a lack of additivity. Likewise, Kv1.2/Kv1.6 heteromers could be formed, that can be blocked by either TiTX or α-DTX. In this context, the ability of α-DTX and MgTX to additively enhance DA release might be due to the simultaneous blockade of Kv1.2/Kv1.3 heteromers and of Kv1.2/Kv1.6 heteromers. New experiments would be required to compare the pharmacological sensitivity of monomers and heteromers in a heterologous expression system. Our results are in line with a previous demonstration that 4-AP, a broad-spectrum I_A_-type potassium current blocker, can enhance DA release [27]. Although Kv1 channels have been demonstrated to be expressed in varicose-like structures in the ventral striatum and various basal ganglia nuclei [65], their presence in DA neurons and specific expression in the axon terminals of DA neurons has not received much attention. However, in a recent study performed in mice, DA neurons have been shown to contain Kv1.1, Kv1.2, Kv1.3 and Kv1.6 mRNA. Furthermore, Kv1.1, Kv1.2 and Kv1.6 immunoreactivity was found in TH-positive axonal varicosities in the dorsal striatum [56]. Additional studies evaluating more directly the localization of Kv1 channels in dopaminergic axons would be useful to determine whether the channels are directly localized at release sites or are found farther along the axonal membrane, separate from the active zone.

Compatible with the results of experiments evaluating the effect of Kv1 antagonists alone, we found that the same antagonists and antagonist combinations that enhanced DA overflow also were effective at reducing the effectiveness of the D2 agonist quinpirole to inhibit DA overflow. The ability of a combined application of TiTX and MgTX to additively reduce the presynaptic effect of quinpirole is puzzling considering their lack of additivity in enhancing DA overflow when used in the absence of quinpirole. Although speculative, it is possible that the D2 receptor preferentially regulates the function of Kv1 monomers. Here again, additional experiments using a heterologous expression system would be useful to clarify this issue. Nonetheless, based on our experiments, we conclude that Kv1.2, Kv1.3 and perhaps Kv1.6 are key regulators of presynaptic D2 receptor function and that D2 receptor activation on dopaminergic axon terminals could perhaps lead to an enhancement of Kv1 channel function. Our results are consistent with previous work showing that D2 receptor activation regulates a 4-AP-sensitive potassium current in DA neurons [66] as well as in other cell types [67], [68], although these measurements were made at the cell body level.

Our finding that Kv1 potassium channel blockers reduce the relative effectiveness of presynaptic D2 receptors can be interpreted in two different ways. A first possibility is that Kv channel regulation through the D2 receptor is directly required for a substantial proportion of presynaptic D2 receptor function. For example, a facilitation of the voltage-dependent activation of these channels in response to D2 receptor stimulation could lead to shortening of the action potential in the axon terminal, leading to reduced opening of voltage-dependent Ca^2+^ channels and DA release. A second possibility is that Kv1 channels are not directly regulated by the D2 receptor, but act instead as a gating mechanism, influencing the impact of D2 receptor activation on an alternate target, such as voltage-dependent Ca^2+^ channels. Considering on the one hand that Kv channels play a role in maintaining membrane potential and modulating electrical excitability in neurons [35], and on the other hand that G-protein inhibition of N-type calcium channels is voltage-dependent [69], it may be hypothesized that a blockade or reduction in Kv1 channel function in axon terminals causes a modest depolarization of DA terminals, which could reduce the ability of the D2 receptor to inhibit N-type calcium channels. Arguing against such a hypothesis of terminal depolarization, we found that direct depolarization of terminals with elevated potassium ACSF failed to significantly reduce D2-mediated inhibition of DA overflow. Our previous demonstration that D2 receptor activation inhibits exocytosis evoked independently of calcium channels in cultured DA neurons through a mechanism blocked by 4-AP [70] also speaks in favor of the hypothesis of a more direct role of Kv channels. Moreover, it has been proposed that other terminal G-protein-coupled receptors can inhibit neurotransmitter release by acting directly through the regulation of potassium channels [71], [72], [73], [74]. However, further experiments are required to evaluate whether the D2 receptor can directly regulate the function of Kv1 channel subtypes in DA neurons. Whether activation of Kv1 channels in response to D2 receptor stimulation leads to spike narrowing or other changes in terminal excitability will also need to be directly investigated.

A possible trivial explanation of the ability of Kv1 channel blockers to reduce D2-mediated inhibition of DA release is that the relative decrease in the effect of quinpirole in the presence of the Kv1 blockers may simply result from the fact that the baseline amount of release is increased in the presence of the toxins. There would thus be a decrease in relative but not absolute DA release. To address this possibility, we re-examined our data to look at absolute levels of DA in a subset of experiments. We find that in fact, in the presence of the Kv1 blockers, both the relative and the absolute levels of DA release are significantly reduced in response to quinpirole, thus validating our approach and interpretation. For example, in control experiments without any antagonist, the absolute decrease of evoked DA release induced by 1 µM quinpirole was of 0.69±0.09 µM (n = 11). In comparison, in the experiments with TiTX, the absolute decrease in DA release in response to quinpirole was 0.48±0.08 µM (n = 6), a value significantly smaller than in control experiments (p<0.05). Similarly, in experiments with 4-AP, the absolute decrease of evoked DA release induced by quinpirole was 0.35±0.04 µM (n = 6), again a value significantly smaller that control (p<0.05).

In summary, our work underlines an important role for Kv1 potassium channels in regulating DA release as well as in regulating the rapid inhibition of DA release in response to presynaptic D2 receptor activation in dopaminergic axon terminals of the dorsolateral striatum. By identifying a critical family of ionic channels regulating DA release from axon terminals, our findings may help to suggest novel strategies to regulate DA release in the context of disease.
